# Clinical features of hereditary angioedema and warning signs (H4AE) for its identification

**DOI:** 10.1016/j.clinsp.2022.100023

**Published:** 2022-03-19

**Authors:** Pedro Giavina-Bianchi, Marcelo Vivolo Aun, Juliana Fóes Bianchini Garcia, Laís Souza Gomes, Ana Júlia Ribeiro, Priscila Takejima, Rosana Câmara Agondi, Jorge Kalil, Antonio Abilio Motta

**Affiliations:** Divisão de Imunologia Clinica e Alergia, Faculdade de Medicina (FMUSP), Universidade de São Paulo, São Paulo, SP, Brazil

**Keywords:** Hereditary angioedema, C1 inhibitor deficiency, Warning signs, Laryngeal angioedema, Abdominal pain, Acronym, HAE:, Hereditary Angioedema, C1INH:, C1 inhibitor, C1INH-HAE:, HAE with C1INH deficiency, HAAAAE (H4AE):, Hereditary, recurrent Angioedema, Abdominal pain, Absence of urticaria, Absence of response to antihistamines, Estrogen association, pdC1-INH:, plasma-derived C1-INH concentrate

## Abstract

**Objectives:**

The study describes a case series of hereditary angioedema with C1 Inhibitor Deficiency (C1INH-HAE) in order to corroborate six clinical warning signs “HAAAAE (H4AE)” to enable early identification of this disease.

**Methods:**

The authors analyzed the C1INH-HAE cohort to analyze the clinical aspects of the present study's patients and corroborate the six clinical warning signs of the Hereditary Angioedema Brazilian Guidelines. Data regarding demographics, the onset of disease, time to diagnosis, frequency of attacks per year, organs involved, triggers, crisis duration and their outcomes, and disease treatment were collected. Then the authors developed an acronym, H4AE, to help healthcare professionals remember the warning signs.

**Results:**

The authors included 98 patients in the study, with a mean age of 38.1 years, 67.3% being female, and 75.3% with a family history of HAE. HAE diagnosis was delayed, on average, 13.7 years after its initial manifestation. Exploratory laparotomy was reported by 26.9%, and orotracheal intubation by 21.3% of the present study's patients; 61.3% and 30.3% of them were admitted at least once in the hospital and in the intensive care unit, respectively. The authors constructed an acronym “H4AE” with the six warning signs of HAE: Hereditary, recurrent Angioedema, Abdominal pain, Absence of urticaria, Absence of response to antihistamines, Estrogen association.

**Conclusion:**

C1INH-HAE is still underdiagnosed and associated with high morbidity. The study showed clinical features of this disease, corroborating the warning signs, which may be useful in raising awareness and improving the diagnosis of C1INH-HAE. The authors suggest the acronym “H4AE” to remind the warning signs.

## Highlights

• ""H4AE"": warning signs for hereditary angioedema identification.
• Hereditary angioedema is unknown and underdiagnosed.
• Hereditary angioedema has high morbidity and may be fatal.

## Introduction

Hereditary Angioedema (HAE) is characterized by recurrent episodes of subcutaneous and submucosa edema, which are induced by bradykinin. The disease is rare and still unknown, and is underdiagnosed by many health professionals. HAE accounts for a minority of cases of all types of angioedema, and its prevalence was initially estimated to be 1:10,000 to 1:50,000.[1], [2], [3], [4], [5], [6] However, new endotypes of this disease have been described, making its prevalence more common than previously thought.^7^^,^^8^ The first biochemical defect associated with HAE was C1 inhibitor (C1INH) deficiency.^9^ Later, other genetic mutations were identified, and HAE was classified in HAE with C1INH deficiency (C1INH-HAE) and HAE with normal C1INH.^8^

The long time elapsed between the onset of symptoms and diagnosis, as well as the access to therapy, increases the risk of death from laryngeal angioedema and disease-related morbidity, affecting the quality of life of patients and their families.^1^^,^^10^ HAE patients visit several physicians before receiving a correct diagnosis, and many of them are misdiagnosed.^11^ Health care professionals should be aware of HAE clinical presentation and screening laboratory tests. In addition, allergy and immunology specialists must be updated with information on the diagnosis and management of patients with HAE.

The two most severe clinical manifestations of HAE are related to laryngeal and intestinal angioedema. Patients who are not properly treated have estimated mortality rates of 25% to 40% due to asphyxiation by laryngeal angioedema.^3^^,^^12^ In the United States, HAE accounts for 15,000‒30,000 emergency room visits per year. These visits often lead to hospitalization and admission to intensive care units.^13^ Incapacitating intestinal angioedema is another major manifestation of HAE, and the intestine can be the main or only organ system involved in an attack. Such patients are often misdiagnosed as having acute surgical abdomen and undergo unnecessary explanatory surgeries.^14^

The present study shows the clinical features of a cohort of HAE, analyzes the relevance of warning signs for the early identification of this disease and suggests an acronym HAAAAE (H4AE) in order to memorize the warning signs.

## Methods

The authors analyzed the C1INH-HAE cohort to assess the clinical aspects of the present study's patients and corroborate the six clinical warning signs, which had been suggested in the 2011 Brazilian Hereditary Angioedema Guidelines: Hereditary, recurrent Angioedema, Abdominal pain, Absence of urticaria, Absence of response to antihistamines, Estrogen association.^15^ Then the authors developed an acronym, HAAAAE (H4AE), to help healthcare professionals remember the warning signs. Only patients with C1INH-HAE, all of them with confirmed decreased C1-INH antigenic level and/or functional activity, were included in the study. Many patients also had the genetic diagnosis of C1INH-HAE. The study was approved by the Research Ethics Committee of the *Hospital das Clínicas* of the School of Medicine University of São Paulo (CAPPesq), CAAE 36022520.0.0000.0068.

This was a cross-sectional study assessing the electronic medical records of C1INH-HAE patients from January 2010 to December 2020. Data regarding demographics, the onset of disease, time to diagnosis, frequency of attacks per year, organs involved, triggers, crisis duration and their outcomes, and disease treatment were collected. To obtain missing data, patients were interviewed if needed.

All continuous variables were expressed as means and their standard deviation. Comparisons between groups were made with the unpaired sample *t*-test. Categorical variables were presented as numbers and percentages, being analyzed by Fisher's exact test. Analysis was performed with GraphPad Prism software (version 6.0a), and *p* values of less than 0.05 were considered significant.

## Results

**Fig. 1:**
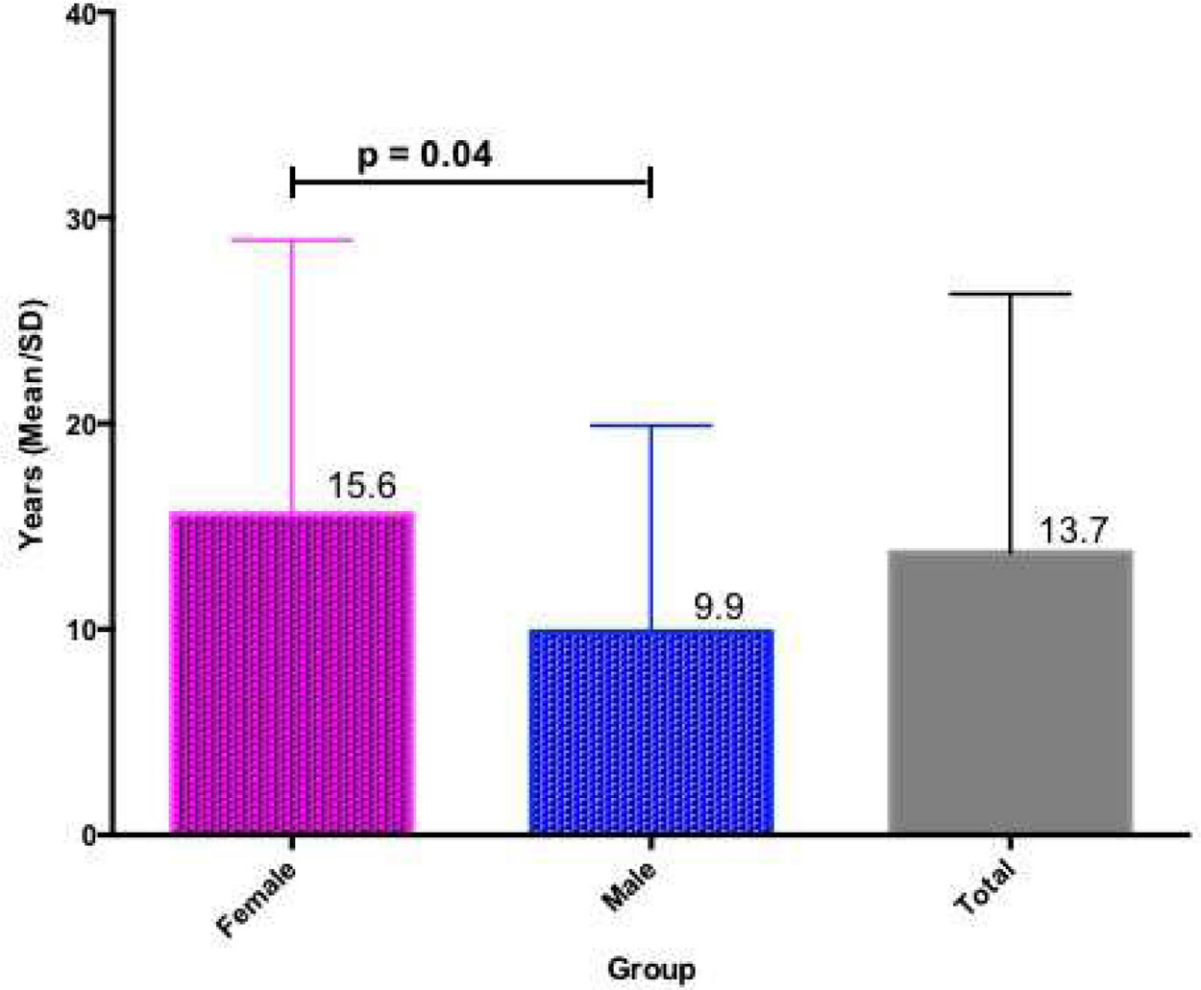
Delay in hereditary angioedema diagnosis. Time in years from the first manifestation to the diagnosis.

The authors included 98 patients in the study, with a mean age of 38.1 (SD = 14.9) years, 67.3% being female, 94.9% with C1INH-HAE Type I, and 75.3% with a family history of HAE. The mean age at onset of symptoms was 12.7 (SD = 10.8) and the mean time to diagnosis was 13.7 years (SD = 12.6) (Table 1). There was a predominance of females in the present case series, and the diagnosis was more delayed in women than in men (p = 0.04) (Fig. 1).

**Table 1:** Clinical features and treatment of hereditary angioedema according to sex.

	Female	Male	Total	*p*-value
n	66	32	98	‒
Age (mean in years)	40.9	32.3	38,1	< 0.01
Age at symptoms onset (mean in years)	13.6	10.8	12.7	0.3
Delay in diagnosis (mean in years)	15.6	9.9	13.7	0.04
Family history of HAE (%)	73.4	84.4	75,3	0.3
Attack rate (annualized average)	12.2	9.0	11,3	0.3
Attack duration (mean in hours)	86.4	70.4	81.2	0.05
Long-term prophylaxis (%)	68.2	75	70.4	< 0.01
Exploratory laparotomy (%)	33.3	10.5	26.9	0.07
Orotracheal intubation (%)	20.0	24.0	21.3	0.77
Hospital admission (%)	62.0	60.0	61.3	1.0
Intensive Care Unit admission (%)	28.0	34.6	30.3	0.6

Before starting treatment, the patients had an average of 11.3 (SD = 13.2) attacks per year that lasted 81.2 (SD = 35.2) hours, and 70.4% of them, mainly men (*p* < 0.01%), were on long-term prophylaxis (Table 1). Current or previous use of danazol and tranexamic acid were observed in 65.3% and 34.7% of these patients, respectively. Women had longer HAE attacks, but the difference was not statistically significant, while men were more subjected to long-term prophylaxis (*p* ≤ 0.01).

**Fig. 2:**
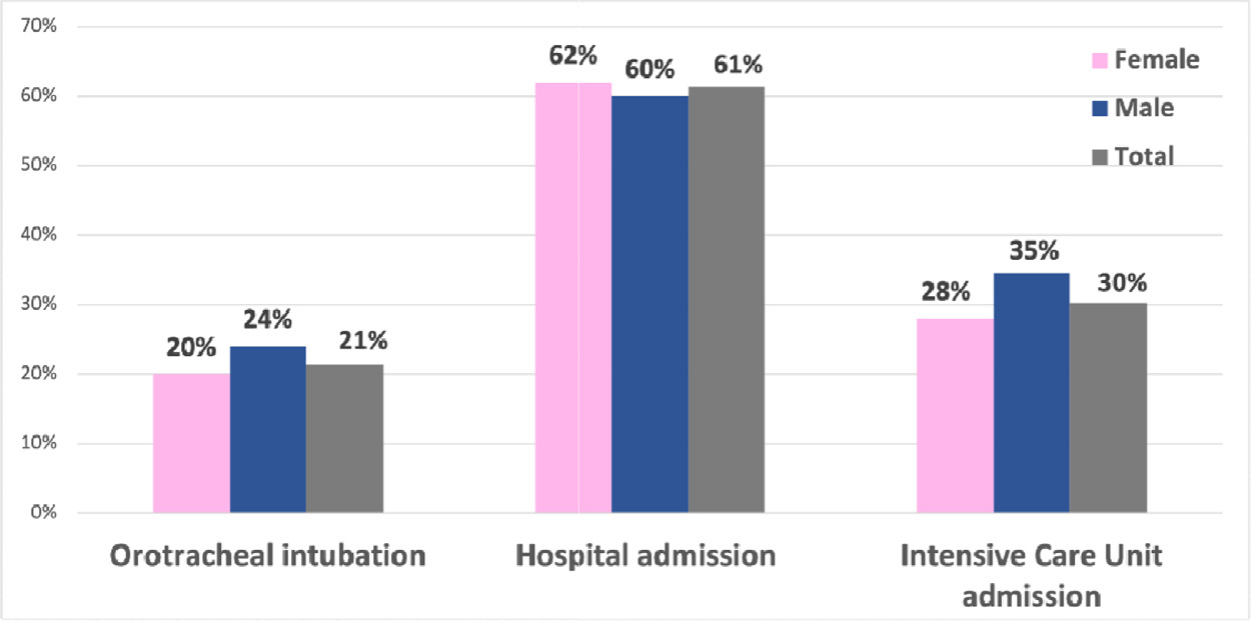
Severity of hereditary angioedema attacks.

The main sites affected in descending order of frequency were extremities (97.5%), face (87.8%), abdominal (87.6%), genitalia (57.6%), larynx (46.9%), and tongue (36.9%). Spontaneous attacks were reported by 81.2% of patients and the main triggering factors reported were stress (72.2%), trauma (67.5%), dental procedures (34.8%), surgical procedures (21.1%), infections (16.4%), and temperature variations (14.7%). Exploratory laparotomy was reported by 26.9% and orotracheal intubation by 21.3% of the present study's patients; 61.3% and 30.3% were admitted at least once in the hospital and in the intensive care unit, respectively (Fig. 2).

Among women, 29.6% reported worsening of the disease in the perimenstrual period and 31% with exogenous estrogen intake. At least one pregnancy was reported by 72.2% of female patients, 15.4% with improvement, and 46.2% with worsening of HAE during pregnancy. Of all deliveries, 68% were cesarean sections indicated by the obstetricians who were assisting the patients.

**Fig. 3:**
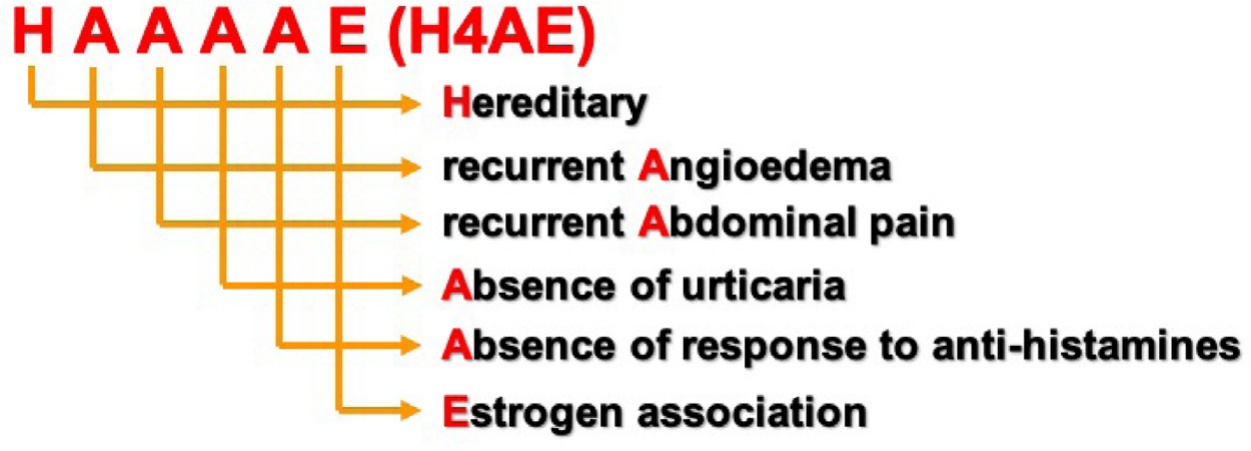
Warning signs.

The authors constructed an acronym to facilitate remembering the six clinical warning signs of the Brazilian Hereditary Angioedema Guidelines. The acronym is made from the initials of Hereditary Angioedema (HAE), inserting four “As” between the “H” and the “E” (Fig. 3). HAAAAE (H4AE) means Hereditary, recurrent Angioedema, Abdominal pain, Absence of urticaria, Absence of response to antihistamines, Estrogen association.

## Discussion

Since the first Brazilian Hereditary Angioedema Guidelines were published in 2011 ^15^ and then updated in 2017,^1^ the body of knowledge regarding HAE has increased, more patients have been diagnosed, and their management has improved. Despite advances in the management of the disease, the present study shows that HAE diagnosis is still very delayed, on average 13.7 years after its initial manifestation, especially in women. Traditionally, women seek medical assistance more frequently and earlier than men, and perhaps they are seen with fewer symptoms at the first clinical manifestations of HAE when the diagnosis is more difficult to be done. Another possible contributing factor is that abdominal crises can be misdiagnosed as other gynecological conditions that are prevalent in women. Finally, the present study's male patients had a higher rate, although not statistically significant, of family history of HAE, facilitating their diagnosis.

The delay in the HAE diagnosis highlights the importance of developing methodologies and teaching instruments to increase awareness and identification of this disease. The present study describes a case series of HAE and analyzes if the 'patients' clinical manifestations are compatible with the warning signs proposed in the 2017 Brazilian HAE Guidelines^1^. In addition, we suggest an acronym, H4AE (HAAAAE), to remember these warning signs. Reminding the four “As” from the American Academy of Asthma, Allergy, and Immunology may be an easy way to memorize that there are four “As” in the acronym.

The “H” of “H4AE” is “Hereditary”. HAE has an autosomal dominant inheritance and, therefore, it has a risk of affecting 50% of the 'patients' offspring. In the present study's case series, a family history of angioedema was observed in 75.3% of the patients, a result in agreement with the literature. Previous studies had shown de novo mutation in 20% and asymptomatic condition in 5% of HAE patients.^3^^,^^6^

The first and the second “As” of “H4AE” are “Recurrent Angioedema” and “Abdominal pain”, respectively. HAE is a rare, life-threatening, disabling disease, and patients live with the fear of having laryngeal angioedema that can lead to death by asphyxiation. Furthermore, abdominal attacks are very troublesome, and patients often inadvertently undergo exploratory laparotomies. More than 95% of the present study's patients had attacks on the extremities and more than 85% on the face and abdomen. Half of the patients had at least one episode of laryngeal edema, and 26.9% were mistakenly submitted to laparotomy. These results are consistent with previous scientific evidence.^3^^,^^6^

The third and the fourth “As” of “H4AE” are “Absence of urticaria” and “Absence of response to antihistamines”, respectively. The present study's patients denied urticaria and referred that their angioedema attacks did not improve with antihistamines, corticosteroids or epinephrine, drugs appropriate to treat histaminergic angioedema. Seventy percent of the patients were doing or had previously done long-term prophylaxis, mainly with danazol, and only 25% of them had used plasma-derived C1-INH (pdC1-INH) concentrate at least one time. Access to pdC1-INH and icatibant is still precarious in Brazil, but we advise the present study's patients in crisis to seek the emergency room of our hospital, where icatibant is available.

The “E” of “H4AE” is “Estrogen association”. Scientific evidence shows that C1INH-HAE may be more severe in females.^16^^,^^17^ However, estrogen association is stronger in HAE with normal C1INH.^7^ The promoter region of the FXII gene contains an estrogen-responsive element, and increases in FXII mRNA transcription in response to estrogen have been demonstrated.^18^ It is likely that estrogen also contributes to the regulation of B2 bradykinin receptor expression, modulates the kallikrein-kinin cascade, and reduces C1-INH levels.^19^^,^^20^ In the present study's case series, there was a female predominance, but the disease was not more severe in women. One-third of them reported disease worsening with exogenous estrogen intake.

## Conclusions

In this study, the authors show the present study's case series in C1-INH-HAE.

It is important to stress that HAE is still underdiagnosed and associated with high morbidity and mortality.

HAE diagnosis was delayed on average 13.7 years after its initial manifestation.

The disease was more prevalent but not more severe in women.

The study showed the relevance of warning signs in the identification of C1-INH-HAE, which may be useful in raising awareness and improving the diagnosis of this disease.

The authors suggest an acronym “H4AE” to remind the warning signs.

## Authors' contributions

All authors participated in the design of the present study and in the analysis of its results. All authors read, approved, and consented to the publication of this manuscript.

## Conflicts of interest

The authors declare no conflicts of interest.
